# Monthly Summary

**Published:** 1885-03

**Authors:** 


### MONTHLY SUMMARY.

---

THE REMOVAL OF STAINS FROM THE TEETH CAUSED BY ADMINISTRATION OF MEDICINAL AGENTS AND THE BLEACHING OF PULPLESS TEETH.—*By A. W. Harlan, M. D., Chicago, Ill.* (*Read in the Section of Oral and Dental Surgery of the American Medical Association, May*, 1884.)—GENTLEMEN—A large number of remedial agents administered by physicians temporarily stain the teeth, but in looking over the list I find there are but few which may be said to permanently stain them. The mineral acids—nitric, sulphuric, hydrochloric, and other acids of this nature, if used for any length of time, may discolor the teeth and likewise have a deleterious effect on them; yet it cannot be said that such agents stain the teeth so that any particular method should be desired for restoring their natural appearance. The vegetable series may likewise be dismissed. The tannates and astringents generally do not permanently stain the teeth. The muriated tincture of iron, and in fact all the ferrum preparations, with the single exception of ferrum dialystum, do stain the teeth; yet it is comparatively easy to remove such stain, by the use of dentifrices and tooth pastes, when of recent occurence. Occasionally it may be necessary to polish the teeth with wooden or leather points charged with finely pulverized emery or levigated pumice; following these, powdered Arkansas stone and precipitated chalk, incorporated with a thick solution of white castile soap, should be used. The stains produced by hydrastis, aloes, tobacco, rhubarb, ink, infusion of saffron, pinus canadensis, carmine, catechu, hydriodic acid, tincture of iodine, and kindred agents, are of temporary duration in nearly all cases. Not so with tobacco, when the enamel has been fractured, or the masticating surfaces of teeth have been deprived of their enamel by wear. In such cases there is no remedy for the stain, when it disfigures, except to cap the teeth with gold. This last condition it is seldom found necessary to treat, as in such cases generally it is only imperative when the ends of the teeth have become sensitive to thermal change. Staining of the teeth as a result of age, it is not the province of this paper to treat. The single substance, which I have found to stain the teeth permanently, is nitrate of silver. Solutions are frequently used in the mouth and throat of too great strength. Many times the powdered nitrate is used to dust diseased mucous membranes, and the teeth then suffer. Occasionally, dental surgeons use the solid stick on the walls of sensitive teeth, and also to arrest incipient caries. When carelessly used it may stain other teeth than those operated upon. These cases are the ones we are called upon to treat. It matters not how the stains are acquired, they must be removed, and no amount of polishing, without injury to the external surfaces of the teeth, will remove such stains. The agents which may be used for removing such stains are not numerous. Cyanide of potassium, iodide of potassium, tincture of iodine, and the liq. amm. fortior are all recommended. There are other substances which may be used to remove fresh stains, notably solution of chloride of sodium if used immediately. I recommend as the neatest, safest, and most certain method, the adjustment of the rubber dam; then dry the teeth, and paint three or four of them with the compound tincture of iodine, allowing it to dry; then moisten the surfaces of the teeth with the stronger liquor ammonia for two or three minutes, and wash the surfaces with peroxide of hydrogen. The stains will have disappeared in consequence of the chemical change having taken place resulting in the formation of iodide of silver and nitrate of ammonia. The teeth should then be polished in the usual manner.

BLEACHING TEETH.

It is not intended that a history of various methods of bleaching teeth in use should be presented for consideration at this time; but it is necessary to remark that most of them are faulty and very few useful in all cases. In order to bleach a pulpless tooth the operator must first fill the root at least one-third its length. All decay should be removed and the fragments of the pulp in the fine angles of the outline of the living pulp should also be removed. Discolored dentine if hard need not be cut away. With the rubber dam adjusted over the adjacent teeth, including the one to be operated upon, the cavity is thoroughly washed with $H_2O_2$ repeatedly and then carefully dried by using the hot blast from a powerful bulb syringe. A small quanity of choride of alumina is placed within the cavity, and it is moistened with peroxide of hydrogen and allowed to remain five minutes. The deliquesced $Al_2Cl_6$ is carefully washed out of the cavity with a clear solution of sodæ biboras $Na_2B_4O_7\,10H_2O$, and the cavity thoroughly dessicated. In the vast majority of cases the tooth will have returned to its normal color. The cause of the change in color is owing to the complete oxidation of the infiltrate into the tubules and the destruction of the staining of the contents of the tubules. The tooth should not be bathed in creosote, carbolic acid, alcohol, or any other substance capable of coagulating albumen. Where the operator has reason to suspect that any such agent has been introduced into the cavity, it should first be washed with the clear solution of sodæ biboras, and then followed by the above-mentioned method. The rapid liberation of chlorine from $Al_2 Cl_6$ in the presence of $H_2 O_2$, resulting in the formation of H-Cl and $H_2 O$, leaving unsatisfied O and Cl accounts for the speedy destruction of the coloring matters within the pulpless tooth. In order to maintain the color an oxy-chloride of suitable color should be used to fill the remainder of the pulp canal, and as large a portion of unfilled cavity as judgment will indicate; thirty to fifty minutes should be given for the hardening of the oxy-chloride. The cavity should be filled with gold immediately. It is not wise to allow saliva or other liquids to come in contact with the oxy-chloride after it is introduced into the tooth. It jeopardizes the permanency of every case when oxy-chloride becomes moist, on account of the speedy absorption of fluids by the freshly hardened filling.

---

CHOICE OF TEETH FOR EXTRACTION FOR THE PURPOSE OF UNIFORMITY.—The physician is not always consulted relative to the best course to adopt in regard to the teeth, but he cannot afford to be ignorant of the peculiarities of this important part of the anatomy. When the teeth are too much crowded in the jaw, it is frequently desirable for æsthetic and hygienic purposes to draw some of them, and the question which to draw when there is a choice has, it appears been a constant source of discussion among dentists. Dr. Perry, in order to throw some light on the subject, has tabulated 7,277 extractions for disease, and finds that 2,823 of these were first permanent molars, 737 were first bicuspids, and 944 were second bicuspids. As the statistics show that more first molars are lost than bicuspids, we should, as a general rule, take out the first molar were the choice must be, as is usually the case, between this and a bicuspid.—*The Weekly Medical Review.*

---

JAPANESE TEETH.—Before the Odontological Society of Great Britain (Dec. 1, 1884), Dr. St. George Elliott exhibited and presented to the museum some very curious artificial dentures of Japanese manufacture. These people had derived most of their technical and scientific knowledge from the

Chinese, but, in this matter, they were far in advance of their teachers; for, whilst the latter could only carve a row of incisors and fasten them to the teeth on either side, the Japanese could make thoroughly serviceable dentures, and had been acquainted with the method of fixing them by suction for about two hundred years. The teeth were mounted on hard wood, those in front being made from quartz pebbles carefully ground down, but the process of mastication was performed by copper nails which occupied the place of the molars. One of the dentures had been in use for fifteen years. Dr. Elliott gave a very interesting account of the way in which they were made and fitted.

---

THE HYPODERMIC SYRINGE.—*By J. S. Geigley, M. D., Lewiston, Ill.*—In Dr. Bumstead's communication to the Clinical Society, in the last number of the MONTHLY, he made some observations on hypodermic injections, and, as this is a matter that is not very frequently discussed, I think I may be pardoned for delivering myself of a few thoughts on the subject. I've often been surprised at the ignorance or carelessness manifested in the use of this little instrument by many physicians, men, too, who were capable and acute in other things. but who seemed to have failed to become thoroughly acquainted with an instrument that is so potent for good, when judiciously used. For my own part, I should feel utterly helpless without a good hypodermic syringe, in the treatment of some of the more malignant malarial affections which we often meet along the river bottoms, not to say anything of the innumerable *algias* over which the hypodermic syringe exerts such a magical influence.

Now what are the requirements for success with the hypodermic method? First of all, we must have a good instrument, the different parts of which should fit so accurately as not to admit of the loss of a single drop of the solution, except through the needle. The piston should fit closely, but not tightly. A good test for a syringe is to withdraw the piston while stopping the opening at the other end with the finger. If the piston rebounds on being released, the instrument can be relied on. Now, next comes the needle. A

good needle is everything, while a poor one is an abomination. If I were given my choice between a good syringe with a poor needle and poor syringe with a good needle, I should choose the latter every time, for I should be much more confident of getting my solution in the right place with it. The needle should be of good size, and not too long. A large needle gives no more pain than a small one, and does not become clogged so easily. The point should be perfectly sharp, with no rough edge, and should have the very slightest bend in the direction of the bevel. An elegant needle has lately been placed on the market, under the name of the "re-enforced hypodermic needle." Now as a good instrument might be rendered worthless in a short time by not having the proper care, perhaps a few words on that subject would not be out of place

The piston should be occasionally removed from the barrel, and the packing thoroughly cleaned and oiled. It is always well enough to keep a drop or two of water above the piston. This keeps the leather from shrinking if the instrument is not used for several days. Always after using the syringe the needle should be thoroughly cleansed. This is important, for two reasons—first, it preserves the potency of the opening and keeps the needle in good order; second, it might prevent infecting some one with a contagious disease. A little dilute acid first, and water afterward, drawn into the syringe, will accomplish the first and prevent the second. If a needle should become clogged so that the obstruction cannot be removed with the wire, or by soaking in dilute acid, it will be found to clear instantly if held in the flame of a spirit lamp, or even a match. True this will draw the temper of the needle, but it can be pretty well restored by plunging it in a glass of cold water, while at a red heat.

Now, as to the points of election for the insertion of the needle: For all ordinary drugs, such as solutions of morphia, atropia, apomorpea, pilocarpine, etc., the backs of the arms, anywhere from the wrists to the shoulders, are preferable on on account of being less sensitive and there being fewer vessels to run the risk of puncture. The same is true of the lower limbs, but, when irritating injections such as acid solutions of quinine, chloral, or ergot, are to be used, they will be found to give the least trouble if injected in the buttocks or on the backs of the shoulders. In hypersensitive persons, some kind of a local anæsthetic will be found useful. I have found that a small piece of ice pressed on the skin for a short time before making the punctures will answer admirably. Another point worth remembering, is, not to use too much strength in picking up the skin in which to make your puncture; for, in an individual with a delicate skin, an unsightly discoloration would be apt to follow, which would be annoying to the patient and embarrassing to the physician.

Now, a few words in regard to the solution usually employed for hypodermic use, and I am done. These should, as a rule, be prepared as used; the compressed hypodermic tablets now on the market enable us to do this with little loss of time and perfect accuracy. If the solution is heated in a spoon over a lamp, before being drawn into the syringe, it will be rendered perfectly aseptic, and, as the solution is more perfect, it is less liable to create local disturbance.

In regard to the hypodermic use of the various drugs, I shall only refer to one specialty, and that is, quinine. In my opinion, five grains, in proper solution, of this drug, given hypodermically, will do more toward breaking up an intermittent or a remittent fever, than twenty grains will do if given by the mouth. Then, too, the cerebral disturbance will not be so great. There is a great saving of the drug. And we will not be depending upon a disordered stomach for the absorption of our quinine.

The solution I use is this:

| | |
|---|---|
| Quiniæ sulph., | 5 grains. |
| Acid. tartaric, | 2½ grains. |
| Aqua, | q. s. ad. 1 drachm. |

This is to be boiled in a test tube over a spirit lamp, until perfectly clear. It will not precipitate on cooling, and, being quite dilute, will not cause abscess, and but very little pain. To break up an intermittent, I usually inject all of the solution, half in each shoulder, two hours before the expected chill. I treat neuralgia of malarial origin in the same way, only adding a quarter of a grain of morphia to the solution.—*Peoria Med. Monthly.*

